# Iron insight: exploring dietary patterns and iron deficiency among teenage girls in Sweden

**DOI:** 10.1007/s00394-025-03630-z

**Published:** 2025-03-04

**Authors:** Anna Stubbendorff, Beata Borgström Bolmsjö, Tomas Bejersten, Eva Warensjö Lemming, Susanna Calling, Moa Wolff

**Affiliations:** 1 Nutritional Epidemiology, Department of Clinical Sciences Malmö, Lund University, Jan Waldenströms gata 35, 214 28 Malmö, Sweden; 2 Center for Primary Health Care Research, Department of Clinical Sciences, Lund University, Malmö, Sweden; 3 University Clinic Primary Care Skåne, Region Skåne, Sweden; 4 Department of Food Studies, Nutrition and Dietetics, Uppsala University, Uppsala, Sweden; 5 Medical Epidemiology, Department of Surgical Sciences, Uppsala University, Uppsala, Sweden

**Keywords:** Sustainable diet, Iron deficiency, Teenage, Adolescent, Nutrient status

## Abstract

**Purpose:**

This observational study examined the relationship between self-reported dietary patterns—omnivore, pescatarian, vegetarian, and vegan—and iron status among Swedish teenage girls. Additionally, we compared the consumption of various food groups in relation to iron status.

**Methods:**

Data were collected from 475 female high school students in Malmö and Lund, Sweden, using questionnaires on dietary habits, iron supplementation, and demographic factors. Participants were classified into dietary groups: 347 omnivores, 38 pescatarians, 27 non-consumers of red meat, 60 vegetarians and 3 vegans. Blood samples were analysed for ferritin and haemoglobin levels to determine iron status. Iron deficiency was defined as ferritin < 15 µg/L, and anaemia as haemoglobin < 110 g/L if < 19 years and < 117 g/L if ≥ 19 years. ANOVA and logistic regression were used to compare biomarker levels and the prevalence of iron deficiency and anaemia across dietary groups.

**Results:**

Omnivores had the highest estimated ferritin levels (19.6 µg/L), which was significantly higher than pescatarians (14.7 µg/L, *p* = 0.03), and vegans/vegetarians (10.9 µg/L, *p* < 0.001). Overall 38.1% of participants were iron deficient. Vegetarians/vegans and pescatarians were significantly more likely to be iron deficient (69.4%, *p* < 0.001 and 49.4%, *p*-value 0.016, respectively) compared to omnivores (30.5%). Lower red meat consumption and higher intake of vegetarian patties and legumes were linked to an increased risk of iron deficiency. Anaemia prevalence (haemoglobin < 110 g/L if < 19 years and < 117 g/L if ≥ 19 years) was 3% across all dietary groups.

**Conclusion:**

This study highlights a higher prevalence of iron deficiency among Swedish teenage girls adhering to plant-based diets. Public health strategies should promote balanced diets that ensure adequate iron intake and absorption while considering environmental sustainability. Regular screening and targeted dietary recommendations are essential for supporting the health of this population.

**Supplementary Information:**

The online version contains supplementary material available at. 10.1007/s00394-025-03630-z

## Introduction

Iron deficiency remains the most prevalent nutritional deficiency worldwide and a leading cause of anaemia, particularly among women and children [1, 2]. Teenage girls are especially vulnerable to iron deficiency due to rapid growth and significant iron losses during menstruation [3]. In the latest national representative dietary survey conducted among teenagers in Sweden, the prevalence of iron deficiency was around 30 percent in 15 and 18 year-old girls [4]. Several prior studies have explored the impact of dietary habits on iron intake and iron status, and highlighted diet as a critical factor to either exacerbate or mitigate the risk of iron deficiency anaemia [5, 6]. Studies have shown that vegan diets often contain higher iron levels compared to omnivorous diets [7–10]. However, findings related to anaemia and iron deficiency in these diets have been inconsistent or contradictory [7, 9–12].

Global dietary habits are key drivers of climate change, responsible for about one third of global anthropogenic greenhouse gas emissions [13, 14]. Reducing consumption of animal-sourced food, especially red meat might reduce climate impact and other adverse environmental impacts from diets [15, 16]. It is estimated that a high-meat diet has four times the environmental impact of a vegan diet in terms of greenhouse gas emissions and land use, nearly triple the negative impact on biodiversity, and double the water use [17]. Shifting towards climate friendly diets, rich in plant-based foods, might mitigate the environmental impact from food. A diet with more plant-based foods and fewer animal-sourced foods has also been shown to help prevent chronic diseases and reduce premature mortality [18, 19]. Consequently, people are being encouraged to eat more plant-based foods, especially in high income countries [20]. In Sweden, national dietary surveys have highlighted shifts in the eating patterns of teenagers, with trends indicating a decrease in meat consumption and a diversification of diets that might affect micronutrient status, including that of iron [4, 21]. Adolescence is a time of rapid growth and physiological development, leading to increased nutritional demands. Poor nutrition during this critical stage can have lasting impacts on overall health and may also affect the health of future children [22].

Few studies have examined the prevalence of iron deficiency among Swedish adolescents. A study conducted in Gothenburg found that 37% of 15–16-year-old girls were iron deficient in 1994 [23]. By 2000, this figure had increased to 45%, possibly due to the removal of iron fortification in flour. In comparison, the national survey Riksmaten Adolescents 2016–2017 reported a prevalence of 29% in the same age group [4].

There is a need for studies of iron status in young girls with various eating patterns. This study assesses iron deficiency among Swedish teenage girls, examining the relationship between their dietary choices and iron status. This paper aims to investigate how dietary patterns, including meat consumption and the intake of discretionary and healthy foods, influence iron status among these individuals. Through this research, we aim to contribute valuable data to the ongoing discussions on adolescent health and nutrition policies in Sweden and beyond, emphasizing the need for targeted dietary recommendations to prevent iron deficiency and support overall health in teenage girls.

## Material and methods

### Study design and subjects

The Iron Insight study, conducted in October 2023, took place at two high schools in Malmö and Lund, situated in southern Sweden. The recruitment process involved inviting all female students from these schools through a combination of public-school area advertisements and information disseminated by teachers. Two events were organized at each school, featuring a general information session followed by data collection. The theoretical sampling frame consisted of 1656 female students, based on the total number of female students enrolled at both schools (841 in Malmö and 815 in Lund). However, the practical sampling frame was limited to the combined seating capacity of the auditoriums, which could accommodate a total of 1300 students across four information sessions. Upon attendance at the information event, the female students were presented with the opportunity to participate and were provided with a QR code linked to a questionnaire, accessible via their mobile phones. The questionnaire covered various aspects, including background characteristics, menstruation, symptoms of fatigue, nutritional supplement use and dietary habits. Following the completion of the questionnaire, participants displayed the confirmation code to the staff, who then proceeded to assess the participants' weight and height. Subsequently, blood samples were obtained.

The inclusion criteria were: being a female high school student in Lund or Malmö, aged 15 years or older, and being post-menarche. Exclusion criteria included participants with inflammatory bowel disease, rheumatoid arthritis, other chronic inflammatory disease, ongoing bacterial infection (e.g. those undergoing antibiotic treatment for urinary tract infection or tonsillitis), and pregnancy. The study design and procedures were approved by the Swedish Ethical Review Authority (Dnr 2023-01088-01), and participants provided written informed consent before inclusion in the study.

### Blood sample collection and laboratory analysis of nutrient status

Non-fasting blood samples (6–8 ml) were collected by trained nurses. Ferritin tubes were centrifuged within four hours after blood draw. The blood samples were stored cold overnight and analysed at the regional clinical chemistry laboratory in Skåne (Lund and Malmö) the following day. Ferritin levels were measures using the Atellica IM Analyzer [24], and haemoglobin levels were measured using the Sysmex XN-10 [25]. The coefficient of variation (CV) was 73.9% for ferritin and 8.0% for haemoglobin. Cut-offs for ferritin were based on guidelines from WHO [2]. The normal range was 15–150 μg/L, thus participants with a ferritin level below 15 μg/L were considered iron deficient. However, in Scandinavia, serum ferritin levels ≤ 30 μg/L are traditionally used to define iron deficiency, and therefore this cut-off was additionally used to analyse the prevalence of iron deficiency based on these reference values [26]. As for iron overload, ferritin concentration above 150 μg/L was used as the cut-off [2]. For haemoglobin, we used reference values sourced from the regional healthcare system, and established normal ranges accordingly [27]. A haemoglobin level of 110–160 g/L was considered within the normal range for participants up to 19 years old, while 117–153 g/L was considered normal for participants aged 19 years or older. Consequently, anaemia was defined as haemoglobin below 110/117 g/L.

### Demographic data and health-related parameters

The webform contained questions about age, school and school branch, and place of living (urban/small town/rural). Participants were asked about their menstrual bleedings, using the validated SAMANTA-questionnaire [28]. Based on the SAMANTA-form, the participants’ perception of their menstrual bleeding intensity, frequency and possible limitations were summed into the SAMANTA-score, ranging from 0 to 10, with higher scores indicating more severe bleeding problems [28]. SAMANTA scores of ≥ 3 are considered indicative of heavy menstrual bleeding. Additionally, participants were asked about fatigue during the last month using an 18-item questionnaire. Participants were also queried regarding their consumption of multivitamins and whether these included iron. Additionally, they were asked about their use of separate iron supplements. Those who reported that their multivitamin contained iron or who indicated that they used an iron supplement were classified as iron supplement users.

Participants had their height and weight measured by trained physicians while awaiting their blood sample collection. Body Mass Index (BMI) was calculated as weight (kg) divided by height squared (m^2^).

### Dietary assessment and analysis of nutrient intake

Dietary habits were assessed through a dietary webform developed for this particular study, covering various aspects of participants' eating patterns. Some questions and answer choices were inspired by the national dietary study “Riksmaten adolescents” 2016–17 [21]. Questions in the webform covered topics such as school meals and the habitual diet classification (e.g., vegan, vegetarian, omnivore, etc.) during the previous year (Supplemental Fig. 1). Additionally, the assessment featured a 14-item Food Frequency Questionnaire (FFQ) covering diet during the previous year, with a specific emphasis on consumption of different types of meat, vegetarian meat-alternatives, discretionary foods, and fruit and vegetables. The eight frequency categories ranged from never/a few times per year to three times or more per day. The comprehensive details of the webform can be found in Supplemental Fig. 1. In addition to the registered frequencies, the data from the FFQ’s were converted into portion sizes and expressed in grams per day, as detailed in Supplemental Table 1. Portion sizes were based on Swedish references [29, 30] and adjusted to align with the requirements of this project. During analysis, a new variable called ‘all types of meat’ was created, comprising the sum of red and processed meat, poultry and fish. Additionally, a binary variable was developed to indicate whether individuals ate more or less than one portion (100 g) of all types of meat per week. To compare participants having an intake above and below the recommended intake of red meat as outlined in the Nordic Nutrition Recommendations (NNR) two other variables were created. One variable indicating a consumption above or below 500 g of red meat as recommended in NNR 2012 [31] and another one with the cut-point of 350 g as recommended in NNR 2023 [32].

### Statistical analyses

Baseline characteristics were summarized as mean values for continuous variables and percentages for categorical variables. Due to the small number of vegans (n = 3), the vegetarian and vegan groups were combined for analysis (n = 63). Participants reporting 'other' diet (n = 4) provided free-text responses, which were reviewed and reassigned to the most appropriate dietary group based on their descriptions. Frequency of consumption of different food groups was captured as eight categories but were merged into four categories before the analyses.

The selection of covariates was based on a directed acyclic graph, DAG (Supplemental Fig. 2). We excluded 8 participants with missing data on SAMANTA. 77 participants with missing values of weight and height were imputed to the overall mean of BMI (21.68). One participant with missing data on iron supplementation was considered non-user. Other missing data were not imputed. Since two participants had missing haemoglobin values, the analyses involving Ferritin included 475 individuals, while those involving haemoglobin included 473 individuals. ANOVA was used to compare biomarker levels between self-reported diets. A multiplicative model was fitted to ferritin and hence ferritin was log-transformed before the analyses and the geometric means from the model presented. Logistic regression was used to compare participants being iron deficient or anaemic between self-reported diets. The statistical models were adjusted for BMI and SAMANTA score as continuous variables, and iron supplementation as a binary variable. Means from the model were estimated using the delta method. Trend analyses were conducted to investigate if more restrictions to the diet led to lower biomarker levels and higher proportions of iron deficiency or anaemia using the same models as above, but with the self-reported diets as a continuous variable instead of as a fixed factor. The variable was coded as follows: Omnivore = 1, non-consumers of red meat = 2, Pescatarian = 3, vegetarian/vegan = 4.

We investigated the association between the consumption frequency of 14 food groups (separately) and the risk of iron deficiency. Logistic regression models were used to calculate odds ratios (ORs) and 95% confidence intervals (CIs) for each food group, as well as for anaemia. Breakfast and school meal consumption were also analysed for potential associations. Additionally, participants were categorized by specific red meat consumption levels using cut-offs of < 500 g/week, < 350 g/week, and < 1 portion/week.

Statistical analyses were performed using Stata SE18 (StataCorp, LLC 2023, College Station, TX). A two-sided *p*-value less than 0.05 was considered statistically significant.

### Sensitivity analysis

Sensitivity analyses were conducted to assess the robustness of our findings. We modelled self-reported diets, biomarker levels and risk of deficiency without adjusting for the covariates iron supplementation, BMI, and SAMANTA score. Additionally, we performed a sensitivity analysis excluding participants with missing BMI data (observed-cases), rather than imputing their BMI values.

## Results

### Demographics and dietary intake

Of the 584 participants who consented to participate in the study, 513 completed the digital diet questionnaire. Of those, 30 participants did not have valid blood sample and eight did not complete the SAMANTA-form. This led to a final study population of 475 individuals, as illustrated in the supplemental Fig. 3.

The mean age was 16.6 years, mean BMI was 21.7 and the majority were non-users of cigarettes and snuff (Table 1). Average SAMANTA score was 3.47, and 53.7% of the participants had a score of 3 or more, indicating heavy menstrual bleeding (possible range 0–10). In this sample, 0.6% self-identified as vegans (n = 3), 12.6% as vegetarians (n = 60), 8.0% as pescatarians (n = 38), and 5.7% as non-consumers of red meat (n = 27). The remaining participants (73.1%, n = 347) considered themself to be omnivores. Girls with iron deficiency had a higher SAMANTA score and a slightly lower BMI. They also had a lower daily meat consumption (all types), while the consumption of vegetarian patties and legumes in grams per day were higher. There were no apparent differences for demographic variables between omnivores, non-consumers of red meat, pescatarians and vegetarians/vegans, except for use of iron supplementation (Table 2). The consumption of iron supplements was 20.6% among vegetarians/vegans, 13.2% among pescatarians, 11.1% among those not eating red meat and 8.9% among omnivores. In the full sample, 53.5% reported eating breakfast every day, and 80.2% reported eating school meals on all school days (Table 1).

**Table 1:** Participants’ demographics and food intake according to iron deficiency and anaemia

	Full sample	Iron deficiency^a^			Anemia^b,c^		
		Yes	No	*p*	Yes	No	*p*
n	475 (100%)	294 (62%)	181 (38%)		459 (97%)	14 (3%)	
Age	16.6 (0.9)	16.6 (0.9)	16.6 (0.9)	0.923	16.6 (0.9)	16.6 (1.0)	0.946
*High school branch*							
Arts (music, theatre, form)	129 (27%)	77 (27%)	52 (29%)	0.085	126 (28%)	2 (15%)	0.518
Humanities or Social Sciences	177 (38%)	120 (42%)	57 (32%)		172 (38%)	5 (39%)	
Natural sciences	158 (34%)	89 (31%)	69 (39%)		151 (34%)	6 (46%)	
*Place of residence*							
In a city	342 (72%)	207 (71%)	135 (75%)	0.064	331 (72%)	10 (71%)	0.605
In a smaller town	107 (23%)	65 (22%)	42 (23%)		102 (22%)	4 (29%)	
In a rural area on the countryside	25 (5.3%)	21 (7.2%)	4 (2.2%)		25 (5.5%)	0 (0%)	
BMI (mean)	21.7 (2.7)	21.8 (2.7)	21.5 (2.5)	0.376	21.7 (2.7)	20.8 (1.9)	0.227
< 18.5	42 (8.8%)	22 (7.5%)	20 (11%)	0.420	38 (8.3%)	3 (21%)	0.114
18.5–24	310 (65%)	196 (67%)	114 (63%)		303 (66%)	7 (50%)	
> 25	46 (9.7%)	31 (11%)	15 (8.3%)		45 (9.8%)	0 (0%)	
Unknown	77 (16%)	45 (15%)	32 (18%)		73 (16%)	4 (29%)	
*Smoking cigarettes*							
No	337 (71%)	204 (69%)	133 (74%)	0.534	324 (71%)	11 (79%)	0.756
Yes, sporadically	130 (27%)	84 (29%)	46 (25%)		127 (28%)	3 (21%)	
Yes, daily	8 (1.7%)	6 (2.0%)	2 (1.1%)		8 (1.7%)	0 (0%)	
*Using snuff*							
No	393 (83%)	245 (83%)	148 (82%)	0.715	379 (83%)	12 (86%)	0.942
Yes, sporadically	46 (9.7%)	29 (9.9%)	17 (9.4%)		45 (9.8%)	1 (7%)	
Yes, daily	36 (7.6%)	20 (6.8%)	16 (8.8%)		35 (7.6%)	1 (7%)	
SAMANTA score total (0–10)	3.5 (2.8)	3.0 (2.6)	4.3 (2.9)	< 0.001	3.4 (2.8)	4.8 (2.6)	0.077
3 points or more	255 (54%)	134 (46%)	121 (67%)	< 0.001	242 (53%)	12 (86%)	0.015
Multivitamin users	81 (17%)	59 (20%)	22 (12%)	0.024	79 (17%)	2 (14%)	0.769
Iron supplementation^c^	52 (11%)	38 (13%)	14 (7.7%)	0.078	49 (11%)	2 (14%)	0.668
*Self-reported diet last year*							
Vegan	3 (0.6%)	1 (0.3%)	2 (1.1%)	< 0.001	2 (0.4%)	1 (7%)	0.072
Lacto-vegetarian	8 (1.7%)	4 (1.4%)	4 (2.2%)		8 (1.7%)	0 (0%)	
Lacto-ovo-vegetarian	52 (11%)	16 (5.4%)	36 (20%)		50 (11%)	1 (7%)	
Pescatarian	38 (8.0%)	20 (6.8%)	18 (10%)		37 (8.1%)	1 (7%)	
No red meat	27 (5.7%)	13 (4.4%)	14 (7.7%)		26 (5.7%)	1 (7%)	
Omnivore	347 (73%)	240 (82%)	107 (59%)		336 (73%)	10 (71%)	
Eating breakfast every day	253 (54%)	167 (57%)	86 (48%)	0.064	242 (53%)	9 (64%)	0.403
Eating school meals every school day	380 (80%)	229 (78%)	151 (83%)	0.162	365 (79.7%)	13 (93%)	0.224
Red meat, g/day^d^	37.7 (44.8)	41.6 (45.1)	31.3 (43.7)	0.015	37.3 (43.9)	49.5 (69.3)	0.317
Processed meat, g/day^d^	4.0 (5.2)	4.5 (5.5)	3.1 (4.5)	0.006	4.0 (5.2)	2.9 (5.2)	0.422
Fish, g/day^d^	19.9 (21.5)	21.4 (23.3)	17.5 (17.9)	0.057	20.1 (21.7)	16.1 (11.1)	0.486
Poultry, g/day^d^	34.2 (36.9)	37.0 (36.3)	29.7 (37.6)	0.037	34.3 (37.0)	34.6 (35.5)	0.977
Total meat, g/day^d^	95.7 (81.5)	104.1 (82.8)	82.1 (77.7)	0.004	95.7 (81.1)	103.0 (99.9)	0.742
Vegetarian patties, g/day^d^	32.2 (39.4)	26.0 (32.4)	42.1 (47.1)	< 0.001	32.0 (38.9)	27.2 (34.8)	0.646
Legumes, g/day^d^	23.0 (26.2)	19.7 (21.5)	28.4 (31.8)	< 0.001	22.8 (25.8)	31.9 (38.5)	0.203
Dairy as drink or in food g/day^d^	102 (89.3)	101 (89.8)	103 (88.8)	0.799	102 (89.5)	101 (91.0)	0.985
Dairy on bread g/day^d^	8.9 (8.8)	8.3 (8.4)	9.8 (9.5)	0.072	8.8 (8.7)	12.4 (12.1)	0.139
Vegetables, g/day^d^	104 (57.2)	101 (56.5)	107 (58.1)	0.259	104 (57.1)	107 (61.2)	0.805
Fruit and berries, g/day^d^	72.0 (63.6)	72.5 (59.2)	71.1 (70.4)	0.821	72.6 (63.9)	55.7 (57.4)	0.328
Candy and snacks, g/day^d^	35.6 (29.3)	34.1 (26.2)	38.0 (33.6)	0.157	35.4 (29.4)	40.7 (29.1)	0.505
Coffee and tea, g/day^d^	133 (155)	132 (155)	135 (155)	0.861	135 (156)	93.2 (113)	0.324
Sugar sweetened beverages, g/day^d^	53.6 (66.8)	54.9 (74.6)	51.5 (51.8)	0.586	53.8 (67.5)	49.4 (45.0)	0.808
Drinks w artificial sweeteners g/day^d^	54.4 (97.3)	54.2 (101.0)	54.7 (91.5)	0.963	54.9 (98.5)	40.8 (56.6)	0.594
Red meat < 500 g/week	395 (83%)	239 (81%)	156 (86%)	0.166	382 (83%)	11 (79%)	0.647
Red meat < 350 g/week	274 (58%)	155 (53%)	119 (66%)	0.005	264 (58%)	9 (64%)	0.614
All meat < 1 port/week (100 g)^e^	66 (14%)	25 (8.5%)	41 (23%)	< 0.001	63 (14%)	2 (14%)	0.952
> 500 g fruit and vegetables/day	466 (98%)	292 (99%)	174 (96%)	0.013	450 (98%)	14 (100%)	0.597

^a^Iron deficiency defined as ferritin < 15 μg/L

^b^Anaemia defined as haemoglobin < 110 g/l if age 18 or below, 117 g/l if > 18 years

^c^Two participants have missing values on anaemia (n = 473)

^d^Conversion of food consumption frequencies into g/day is reported in supplemental material

^e^Sum of meat was calculated by summarizing red meat, processed meat, fish, and poultry

**Table 2:** Participants’ demographics according to self-reported diet

	Self-reported diet				
	Omnivore	No red meat	Pescatarian	Vegan/Vegetarian	*p*-value
n	347 (73.1%)	27 (5.7%)	38 (8.0%)	63 (13.3%)	
Age	16.5 (0.9)	16.6 (0.9)	16.7 (0.9)	16.8 (0.9)	0.150
*High school branch*					
Arts (music, theatre, form)	84 (24.9%)	5 (19.2%)	12 (32.4%)	28 (44.4%)	0.029
Humanities or Social Sciences	138 (40.8%)	8 (30.8%)	14 (37.8%)	17 (27.0%)	
Natural sciences	116 (34.3%)	13 (50.0%)	11 (29.7%)	18 (28.6%)	
*Place of residence*					
In a city	247 (71.4%)	21 (77.8%)	30 (78.9%)	44 (69.8%)	0.195
In a smaller town	83 (24.0%)	3 (11.1%)	8 (21.1%)	13 (20.6%)	
In a rural area on the countryside	16 (4.6%)	3 (11.1%)	0 (0.0%)	6 (9.5%)	
BMI	21.7 (2.9)	21.2 (3.2)	22.0 (3.4)	21.7 (2.8)	0.804
*BMI categories*					
< 18.5	29 (8.4%)	4 (14.8%)	4 (10.5%)	5 (7.9%)	0.644
18.5–24	232 (66.9%)	15 (55.6%)	20 (52.6%)	43 (68.3%)	
> 25	29 (8.4%)	4 (14.8%)	6 (15.8%)	7 (11.1%)	
Unknown	57 (16.4%)	4 (14.8%)	8 (21.1%)	8 (12.7%)	
*Smoking cigarettes*					
No	251 (72.3%)	21 (77.8%)	20 (52.6%)	45 (71.4%)	0.211
Yes, sporadically	91 (26.2%)	6 (22.2%)	17 (44.7%)	16 (25.4%)	
Yes, daily	5 (1.4%)	0 (0.0%)	1 (2.6%)	2 (3.2%)	
*Using snuff*					
No	293 (84.4%)	24 (88.9%)	27 (71.1%)	49 (77.8%)	0.328
Yes, sporadically	30 (8.6%)	2 (7.4%)	7 (18.4%)	7 (11.1%)	
Yes, daily	24 (6.9%)	1 (3.7%)	4 (10.5%)	7 (11.1%)	
SAMANTA score total (0–10)	3.5 (2.8)	4.3 (3.7)	3.2 (2.6)	3.2 (2.8)	0.382
3 points or more	187 (53.9%)	15 (55.6%)	19 (50.0%)	34 (54.0%)	0.969
Multivitamin users	57 (16.5%)	5 (18.5%)	4 (10.8%)	15 (23.8%)	0.367
Iron supplementation^a^	31 (8.9%)	3 (11.1%)	5 (13.2%)	13 (20.6%)	0.053

^a^From multivitamins containing iron or separate iron supplementation

Significant differences between all self-reported diet groups were found in 10 out of 14 examined food groups (e.g., meat, fish, vegetables) when comparing the daily intake in grams (Table 3). Omnivores consumed more total meat, specifically red and processed meat, compared to the other groups (Table 3). Vegans/vegetarians consumed more vegetarian patties, legumes, vegetables and fruit and berries. Pescatarians consumed more fish and coffee/tea compared to other groups. Omnivores consumed less drinks with artificial sweeteners than other groups. Some of those who self-identified as being vegetarian/vegan, pescatarian or not eating red meat reported intake of the excluded food groups within the last year (Table 3, Supplemental Table 2). There were no apparent differences regarding breakfast and school meals between the groups of self-identified diets (Table 3).

**Table 3:** Participants’ biomarker status and food intake according to self-reported diet

	Self-reported diet				
	Omnivore	No red meat	Pescatarian	Vegan/Vegetarian	*p*-value^a^
n	347 (73.1%)	27 (5.7%)	38 (8.0%)	63 (13.3%)	
Ferritin (mean, SD)	24.8 (17.5)	19.9 (15.0)	17.8 (10.7)	15.7 (14.1)	< 0.001
Iron deficiency (ferritin < 15 mg/L)	107 (30.8%)	14 (51.9%)	18 (47.4%)	42 (66.7%)	< 0.001
Haemoglobin^b^ (mean, SD)	132.6 (10.6)	130.5 (9.4)	131.8 (10.7)	130.3 (10.3)	0.347
Anaemia^c^ (Hb < 110/1173 g/L)	10 (2.9%)	1 (3.7%)	1 (2.6%)	2 (3.2%)	0.993
Eating breakfast every day	192 (55.5%)	15 (55.6%)	17 (44.7%)	29 (46.8%)	0.408
Eating school meals every school day	277 (80.1%)	23 (85.2%)	32 (84.2%)	48 (76.2%)	0.694
Red meat, g/day^d^	49.5 (46.4)	15.3 (17.0)	3.8 (9.6)	2.4 (13.0)	< 0.001
Processed meat, g/day^d^	5.1 (5.4)	3.0 (4.7)	0.2 (0.6)	0.0 (0.4)	< 0.001
Fish, g/day^d^	22.6 (21.5)	17.0 (16.4)	26.3 (24.8)	2.7 (9.1)	< 0.001
Poultry, g/day^d^	41.5 (35.4)	54.3 (53.7)	8.2 (17.6)	0.9 (4.5)	< 0.001
Total meat, g/day^d^	118.5 (79.0)	89.6 (65.5)	38.5 (30.5)	6.2 (25.1)	< 0.001
Vegetarian patties, g/day^d^	20.4 (22.3)	42.0 (30.2)	60.1 (52.9)	75.4 (62.0)	< 0.001
Legumes, g/day^d^	16.1 (16.9)	25.7 (18.6)	33.7 (19.4)	53.6 (44.6)	< 0.001
Dairy as drink or in food g/day^d^	98.6 (86.7)	92.3 (97.9)	114.7 (90.4)	114.8 (98.9)	0.415
Dairy on bread g/day^d^	8.3 (8.6)	9.6 (8.7)	9.8 (7.9)	11.2 (10.3)	0.108
Vegetables, g/day^d^	96.2 (55.3)	95.3 (53.3)	119.1 (50.5)	137.7 (59.3)	< 0.001
Fruit and berries, g/day^d^	66.0 (57.9)	73.7 (71.5)	88.8 (77.5)	94.2 (74.9)	0.004
Candy and snacks, g/day^d^	35.2 (28.4)	31.3 (21.6)	41.3 (41.8)	35.7 (28.5)	0.564
Coffee and tea, g/day^d^	117.9 (139.9)	115.2 (136.6)	211.6 (209.5)	176.3 (183.3)	< 0.001
Sugar sweetened beverages, g/day^d^	57.0 (72.7)	36.1 (36.7)	41.0 (37.9)	50.0 (53.9)	0.232
Drinks with artificial sweeteners g/day^d^	46.4 (70.7)	70.2 (145.4)	83.1 (164.8)	74.6 (135.0)	0.028
Red meat < 500 g/week	269 (77.5%)	27 (100%)	38 (100%)	61 (96.8%)	< 0.001
Red meat < 350 g/week	153 (44.1%)	23 (85.2%)	37 (97.4%)	61 (96.8%)	< 0.001
All meat < 1 port/week (100 g)^e^	2 (0.6%)	1 (3.7%)	8 (21.1%)	55 (87.3%)	< 0.001
> 500 g fruit and vegetables/day	343 (98.8%)	27 (100%)	36 (94.7%)	60 (95.2%)	0.084

^a^The *p*-value corresponds to the test of whether all self-reported diet groups have the same mean, using ANOVA for continuous variables and Pearson's chi-square test for categorical variables

^b^Two participants have missing values on haemoglobin/anaemia (n = 473)

^c^Haemoglobin below 110 g/L if participant age < 19 years old and 117 g/L for participants ≥ 19 years

^d^Conversion of food consumption frequencies into g/day is reported in supplemental material

^e^Sum of meat was calculated by summarizing red meat, processed meat, fish, and poultry

### Micronutrient status and self-reported diets

**Fig. 1:**
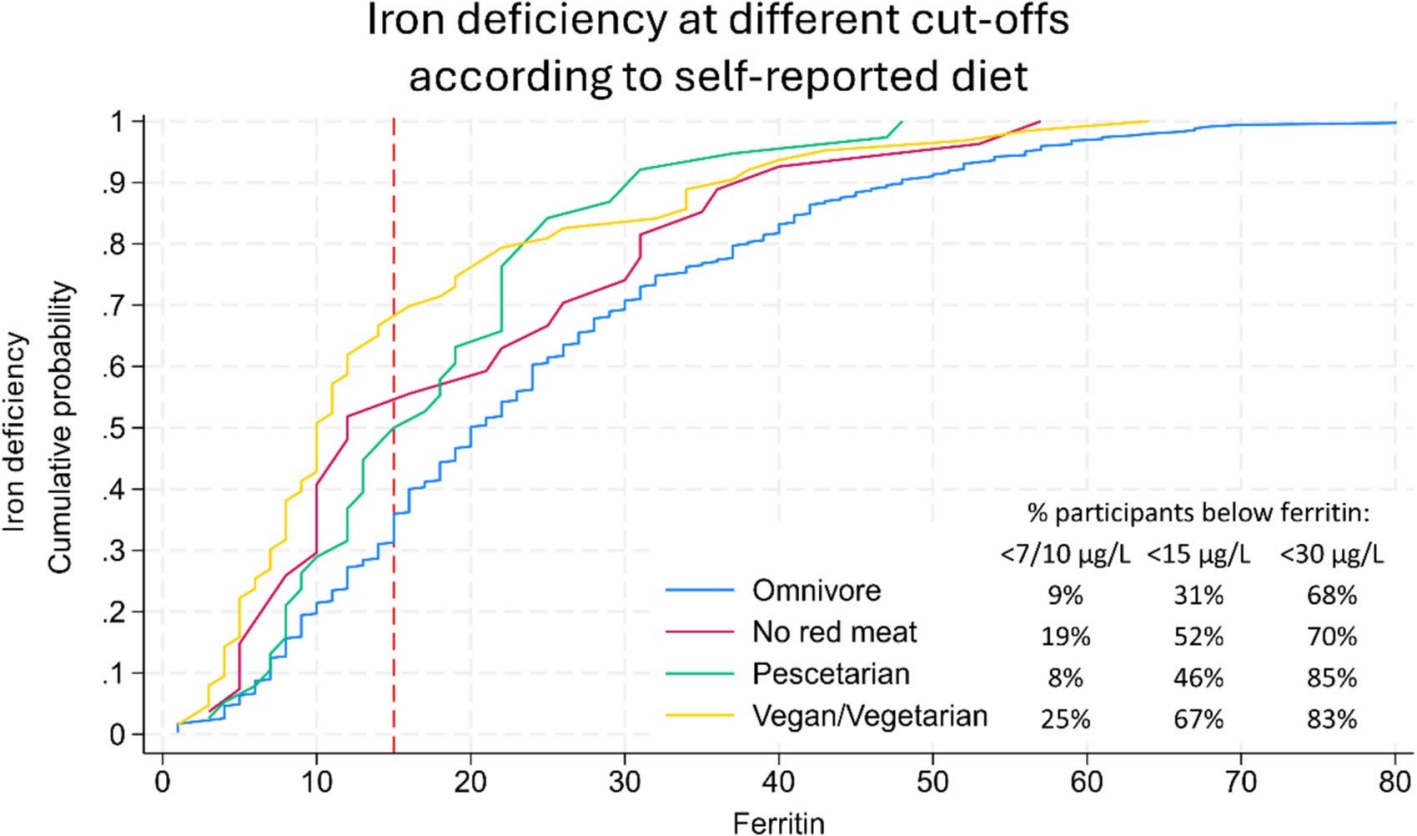
Cumulative distribution functions for each group of self-reported diets

Overall, 38.1% (n = 181) of the participants were iron deficient (ferritin < 15 μg/L), and 3% (n = 14) had anaemia (Hb < 110/117 g/L) (Table 1). The observed prevalences of iron deficiency were 30.8% among omnivores, 51.9% among non-consumers of red meat, 47.4% among Pescatarians, and 66.7% among vegan/vegetarian group (Table 3). The observed ferritin concentrations (mean (SD)) were as follows; Omnivores 24.8 (± 17.5) μg/L, non-consumers of red meat 19.9 (± 15.0) μg/L, pescatarians 17.8 (± 10.7) μg/L and vegetarians/vegans 15.7 (± 14.1) μg/L (Table 3). More restrictions to self-reported diets led to consistently lower ferritin levels as outlined in Fig. 1, displaying the cumulative distribution functions for each group, which was confirmed in the trend analyses. Figure 1 also illustrates that the ferritin cut-off level defining iron deficiency has little impact on the comparisons of the self-reported diets. When using a ferritin cut-off of 7/10 μg/L 11.4% of the total population were classified as iron deficient, and with the cut-off of 30 μg/L it was 72% of the girls that were iron deficient. Despite some significant demographic differences between the two schools, there were no significant differences in ferritin concentrations or haemoglobin levels between them (Supplemental Table 3).

The estimated percentages with iron deficiency, after adjustments for BMI, SAMANTA, and iron supplementation, were 30.5% among omnivores, 48.2% among non-consumers of red meat, 49.4% among pescatarians and 69.4% among vegetarians/vegans (Table 4). Presenting the results according to dietary iron supplementation, omnivores consistently show the highest ferritin and haemoglobin levels, indicating better iron status compared to non-consumers of red meat, pescatarians, and vegans/vegetarians (Table 4). The statistical analyses adjusting for confounders showed that more restrictions to self-reported diets led to statistically significantly higher prevalences of iron deficiency (*p* for trend < 0.001) and ferritin concentration (*p* for trend < 0.001). The odds-ratio (OR) for iron deficiency among vegans/vegetarians was OR = 5.95 (*p* < 0.001) compared to omnivores. Similarly, for pescatarians, the OR was 2.39 (*p* = 0.016) compared to omnivores, while it was non-significant for non-consumers of red meat (OR = 2.27, *p* = 0.06) compared to omnivores. Since the prevalences are dependent on iron supplementation, we reported the estimated prevalences overall and with and without iron supplementation over self-reported diet groups. The statistical analyses showed that vegans/vegetarians had 44.7% lower ferritin levels (*p* < 0.001) and pescatarians had 25.0% lower levels (*p* = 0.03) than omnivores (Table 4). The estimated reduced ferritin levels for non-consumers of red meat were 16.3% (*p* = 0.25) compared to omnivores. There was a positive correlation between BMI and ferritin levels, with each unit increase in BMI corresponding to a 0.02 μg/L rise in ferritin. The estimated marginal means for ferritin (adjusted for BMI, SAMANTA score and iron supplementation) were; Omnivores 19.6 μg/L, non-consumers of red meat 16.4 μg/L, pescatarians 14.7 μg/L and vegetarians/vegans 10.9 μg/L.

**Table 4:** Comparison of self-reported diets and serum levels of ferritin and haemoglobin

	Omnivore	No red meat	Pescatarian	Vegan/ vegetarian
*Estimated mean serum values, mean ratios (%), prevalences (%) and odds ratios (OR)*^*a*^				
Ferritin (μg/L)^b^ Estimated means	19.6	16.4	14.7	10.9
Ferritin Mean ratios (95% CI, *p*-value)	1 (ref)	83.7% (61.8; 113.4, *p* = 0.251)	75.0% (57.9; 97.2, *p* = 0.03)	55.3% (44.9; 68.2 *p* < 0.001)
*Iron deficiency*^*c*^* (estimated prevalences)*				
All	30.5%	48.2% (*p* = 0.059)	49.4% (*p* = 0.016)	69.4% (*p* < 0.001)
Participants taking iron supplement	15.6%	28.6% (*p* = 0.005)	29.6% (*p* = 0.001)	49.7% (*p* < 0.001)
Participants not taking iron supplement	32.3%	50.6% (*p* < 0.001)	51.9% (*p* < 0.001)	71.8% (*p* < 0.001)
*Iron deficiency*^*c*^* Odds ratios*				
OR (95% CI, *p*-value)	1 (ref)	2.3 (1, 5.3, *p* = 0.059)	2.4 (1.2, 4.9, *p* = 0.016)	6 (3.2, 11, *p* < 0.001)
Haemoglobin (g/L)^d^ Estimated means	132.7	130.9	131.5	130
Haemoglobin^d^ Mean ratios (95% CI, *p*-value)	1 (ref)	16.5% (0.3, 993.3, *p* = 0.388)	30.4% (0.9, 1011.7, *p* = 0.505)	6.9% (0.4, 117.6, *p* = 0.065)
*Anaemia*^*e*^* (estimated prevalences)*				
All	2.9%	2.9% (*p* = 0.997)	2.9% (*p* = 0.995)	3.2% (*p* = 0.926)
Participants taking iron supplement	3.6%	3.6% (*p* = 0.414)	3.5% (*p* = 0.388)	3.8% (*p* = 0.248)
Participants not taking iron supplement	2.8%	2.8% (*p* = 0.319)	2.8% (*p* = 0.312)	3.1% (*p* = 0.173)
*Anaemia*^*e*^* Odds ratios*				
OR (95% CI, *p*-value)	1 (ref)	1 (0.1, 8.5, *p* = 0.997)	1 (0.1, 8.1, *p* = 0.995)	1.1 (0.2, 5.3, *p* = 0.926)

^a^Models are based on logistic regression for categorical variables and ANOVA for continuous variables. All analyses are adjusted for BMI, SAMANTA score and iron supplementation. *p*-values are the pairwise test using omnivores as reference

^b^Ferritin was modelled on the log-scale and geometric means are presented

^c^Ferritin below 15 μg/L

^d^Two participants have missing values on anaemia (n = 473)

^e^Haemoglobin below 110 g/L if participant age < 19 years old and 117 g/L for participants ≥ 19 years

The statistical analyses, adjusted for BMI, SAMANTA, and iron supplementation, revealed no statistically significant trend in haemoglobin concentration (*p* for trend = 0.054) or anaemia (*p* for trend = 0.997) associated with increasing dietary restrictions in the self-reported diets. There were no significant differences in haemoglobin levels or presence of anaemia between any of the groups of self-reported diet (Table 4).

### Micronutrient status and food consumption

When investigating the association between consumption of each food group (separately) and iron deficiency, we observed an increased risk with lower consumption of red meat and poultry, as described in Table 5. Consumption of processed meat was associated with a decreased risk of iron deficiency in the group eating 3–4 portions per week (OR 0.41, 95% CI 0.23, 0.75) compared to the reference group (≤ 1 portion per month), but not when comparing the other groups. As for vegetarian patties there was an increased risk of iron deficiency when consuming ≥ 5 portions per week (OR 4.78, 95% CI 2.58, 8.85). Similarly, legume consumption of ≥ 5 portions was also associated with a higher risk (OR 2.47, 95% CI 1.34, 4.54). Participants eating 2–3 portions of fruit or berries per week had a lower risk of iron deficiency compared to the reference group (OR 0.46, 95% CI 0.23, 0.92). For other food groups there were no significant differences in prevalence of iron deficiency. There were no significant differences in consumption of any of the food groups and risk of anaemia in the multiplicative model (Supplemental Table 4). Similarly, the frequency of breakfast intake and school meal consumption had no impact on iron deficiency risk nor anaemia (data not shown).

**Table 5:** Iron deficiency and consumption frequencies of different food groups, analysed with logistic regression, adjusted for covariates BMI, SAMANTA score and dietary iron supplementation

Food groups	Self-reported frequency of intake of different food groups and associated odds ratio for iron deficiency^a^				
	Portions per				
	Month	Week			*p*
	≤ 1	1–2	3–4	≥ 5	
Red meat, n	143	135	120	76	
	1 (ref)	0.27 (0.16, 0.46)			< 0.001
	1 (ref)		0.29 (0.17, 0.5)		< 0.001
	1 (ref)			0.31 (0.16, 0.57)	< 0.001
Processed meat, n	247	85	77	65	
	1 (ref)	0.71 (0.42, 1.2)			0.199
	1 (ref)		0.41 (0.23, 0.75)		0.004
	1 (ref)			0.59 (0.32, 1.07)	0.082
Poultry, n	138	195	108	34	
	1 (ref)	0.31 (0.19, 0.51)			< 0.001
	1 (ref)		0.32 (0.18, 0.56)		< 0.001
	1 (ref)			0.42 (0.19, 0.94)	0.034
Fish and shellfish, n	225	204	39	6	
	1 (ref)	0.89 (0.59, 1.33)			0.568
	1 (ref)		0.61 (0.28, 1.31)		0.205
	1 (ref)			0.74 (0.13, 4.29)	0.741
Vegetarian patties, n	180	141	84	69	
	1 (ref)	0.96 (0.59, 1.56)			0.860
	1 (ref)		1.48 (0.84, 2.59)		0.174
	1 (ref)			4.78 (2.58, 8.85)	< 0.001
Legumes, n	152	162	94	67	
	1 (ref)	1.17 (0.72, 1.9)			0.529
	1 (ref)		1.69 (0.97, 2.94)		0.063
	1 (ref)			2.47 (1.34, 4.54)	0.004
Dairy products in food or as drink, n	52	71	81	271	
	1 (ref)	0.89 (0.41, 1.91)			0.759
	1 (ref)		0.92 (0.44, 1.93)		0.819
	1 (ref)			1.11 (0.59, 2.09)	0.752
Dairy on bread, n	95	110	87	182	
	1 (ref)	0.94 (0.52, 1.7)			0.840
	1 (ref)		0.83 (0.43, 1.57)		0.560
	1 (ref)			1.46 (0.86, 2.48)	0.164
Vegetables	5	22	47	400	
	1 (ref)	0.39 (0.05, 3.08)			0.370
	1 (ref)		0.19 (0.03, 1.37)		0.098
	1 (ref)			0.46 (0.07, 3.05)	0.424
Fruit and berries, n	54	101	106	214	
	1 (ref)	0.85 (0.43, 1.68)			0.633
	1 (ref)		0.46 (0.23, 0.92)		0.027
	1 (ref)			0.57 (0.31, 1.06)	0.077
Candy and snacks, n	35	217	164	58	
	1 (ref)	1.18 (0.54, 2.59)			0.686
	1 (ref)		1.11 (0.5, 2.48)		0.801
	1 (ref)			1.55 (0.63, 3.86)	0.342
Coffee and tea, n	140	89	70	175	
	1 (ref)	1.13 (0.64, 1.99)			0.673
	1 (ref)		0.93 (0.5, 1.73)		0.829
	1 (ref)			1.19 (0.74, 1.91)	0.471
Sugar-sweetened beverages, n	208	182	61	22	
	1 (ref)	0.84 (0.55, 1.29)			0.433
	1 (ref)		1.28 (0.7, 2.33)		0.415
	1 (ref)			0.43 (0.15, 1.21)	0.110
Diet sodas/ beverages, n	278	104	56	36	
	1 (ref)	1.03 (0.64, 1.66)			0.896
	1 (ref)		0.92 (0.49, 1.7)		0.785
	1 (ref)			1.02 (0.49, 2.14)	0.957

^a^Iron deficiency defined as ferritin < 15 μg/L

We observed that among those eating less than 500 g of red meat per week, 39.5% had iron deficiency, compared to those eating more than 500 g where 31.3% were iron deficient (Table 6). When using the cut-point of 350 g we observed that those eating less, 43.4% had iron deficiency, compared to those eating more than 350 g where 30.9% were iron deficient. Those eating less than 1 portion of meat/week (< 100 g of all types of meat) had a prevalence of iron deficiency of 62.1%, compared to 34.2% among those eating more than 1 portion per week. The prevalences of anaemia were similar when comparing different cut-offs.

**Table 6:** Weekly consumption of red meat in relation to presence of iron deficiency and anaemia

	Iron deficiency^a^		Anaemia^b,c^	
	% (n)	*p*	% (n)	*p*
Red meat < 500 g/week	39.5% (n = 156)	0.167	2.8% (n = 11)	0.647
Red meat ≥ 500 g/week	31.3% (n = 25)		3.8% (n = 3)	
Red meat < 350 g/week	43.4% (n = 119)	0.005	3.3% (n = 9)	0.614
Red meat ≥ 350 g/week	30.9% (n = 62)		2.5% (n = 5)	
All types of meat^d^ < 100 g/week	62.1% (n = 41)	< 0.001	3.1% (n = 2)	0.952
All types of meat^d^ ≥ 100 g/week	34.2% (n = 140)		2.9% (n = 12)	

^a^Iron deficiency defined as ferritin < 15 μg/L

^b^Two participants have missing values on haemoglobin/anaemia (n = 473)

^c^Anaemia defined as haemoglobin below 110 g/L if participant age < 19 years old and 117 g/L for participants ≥ 19 years

^d^All types of meat included red and processed meat, poultry and fish. 100 g is considered as one portion

### Sensitivity analyses

The sensitivity analyses largely confirmed the primary results and provided consistent estimated effects, with only minor changes observed in *p*-values. For most analyses, the significance of associations remained unchanged. However, some differences were noted. When examining the associations between self-reported diet, biomarker levels, and the risk of deficiency (total effect), the significant difference in mean serum ferritin levels between pescatarians and omnivores was no longer observed (Supplemental Table 5). In contrast, a significant difference in iron deficiency was identified between non-consumers of red meat and omnivores. The sensitivity analyses excluding participants with missing BMI data, did not alter the results substantially (Supplemental Table 6).

## Discussion

The results of this study underscore the complex interplay between dietary habits and iron status among Swedish teenage girls. Our findings indicate that 38.1% of participants were iron deficient, a significant prevalence that calls for focused interventions. A concerning proportion of both vegetarian and nonvegetarian participants were deficient in iron (serum ferritin < 15 μg/L). Specifically, the prevalence of iron deficiency in vegetarian/vegan participants (69.4%) was more than double compared to the omnivores (30.5%), when adjusting for covariates. In contrast, the prevalence of anaemia did not differ between the groups, although the overall prevalence in all groups was very low (3%).

### Iron deficiency in relation to food items and diet

These recent data collected from teenage girls in two schools in southern Sweden provides a unique opportunity to examine the relationship between diet and iron status in this population. Understanding the dietary determinants of iron status in this population not only helps in addressing iron deficiency more effectively but also enhances our broader nutritional knowledge, thereby supporting public health efforts to improve diet quality among teenagers. Notably, the study highlights the role of diet in influencing iron levels. Participants classified as omnivores exhibited higher ferritin concentrations compared to their counterparts who consumed less or no meat, such as vegetarians, vegans, and pescatarians. This aligns with existing literature that recognizes meat, particularly red meat, as an important source of heme iron, which is more readily absorbed by the body compared to non-heme iron found in plant sources [33]. The observed trend of lower ferritin levels among vegetarians and vegans could be attributed to the lower bioavailability of iron from plant-based sources, partly due to higher levels of phytic acid from whole grains and legumes, which are known to inhibit iron absorption [34]. Non-heme iron absorption can be enhanced by vitamin C and lactic acid-fermented vegetables, and interestingly, in our study, higher consumption of fruit and berries was associated with a reduced risk of iron deficiency. In predominantly plant-based diets, key sources of iron include beans, lentils, peas, nuts, seeds, wholegrain cereals, dark green vegetables, and products made from these ingredients. Excluding meat from the diet does not necessarily lead to a higher consumption of those important food groups.

Our findings suggest that while reducing meat consumption is beneficial from several standpoints, it necessitates careful planning to prevent iron deficiency, particularly in demographics at risk such as teenage girls undergoing rapid growth and experiencing menstrual losses. It has previously been shown in the national dietary survey among adolescents (Riksmaten adolescents 2016–17) that omnivores have the same average intake of dietary iron as those excluding food groups from their diet. However, energy adjusted dietary iron intake was slightly lower among omnivores [21]. In Riksmaten adolescents 2016–17 it was suggested that those taking vitamin and mineral supplements might not be those who need it most, which we could not assume based on the information from this study. In the same study, 26% of the girls aged 18–19 years were iron deficient. Girls with iron deficiency consumed less red meat, including processed meats, than those with the highest iron levels [6]. They also had lower intakes of heme iron. However, the prevalence of iron deficiency was not significantly higher in the small group of girls who excluded meat from their diet compared to those who ate meat. This indicates that their iron requirements might still be met without consuming foods containing the “meat factor” or heme iron. The same study revealed that 50% of girls who had reached menarche had iron deficiency, while only 1% of those who had not yet experienced menarche were deficient. No association was observed between the parents' level of education and the prevalence of iron deficiency. However, among girls born outside of Sweden, 48% had iron deficiency, a significantly higher proportion compared to the 20% observed among girls born in Sweden. Another study, using data from Riksmaten adults 2010–11, reported similar prevalence of iron deficiency (29%) in women of childbearing age (18–44 years) regardless of diet [35]. A study from New Zealand showed that prevalence of iron deficiency was 18.5% among vegetarians and 11% among omnivores (defined as ferritin < 15 μg/L). Despite the smaller magnitude of the difference in this study, anaemia (defined as haemoglobin < 120 g/L) in vegetarian participants (14.8%) was nearly three times greater than nonvegetarian participants (14.8% vs. 5.2%, not statistically significant) [11]. As reported in the present study, a recent Norwegian study among 16–24-year-olds, reported that the risk of anaemia was low regardless of reported dietary preference. However, this study did not measure serum ferritin levels [36]. Few studies have examined the prevalence of iron deficiency among Swedish adolescents. An older study from Gothenburg reported that 37% of 15–16-year-old girls were iron deficient in 1994 (before the removal of iron fortification in flour) and 45% in 2000 (after its removal) [23]. 16 years later, the Riksmaten Adolescents survey suggested a potential decline in iron deficiency among Swedish adolescent girls, reporting a prevalence of 29% [4]. However, our results cannot conclusively address any trend due to its regional focus and the possibility of recruitment bias, as individuals suspecting iron deficiency or anaemia may have been more likely to participate.

When comparing participants based on their consumption of different food groups, we found an increased likelihood of iron deficiency with lower consumption of red meat and poultry. A decreased risk was noted in those consuming moderate amounts of processed meat, but this was not observed in other groups. When comparing iron deficiency levels using the red meat intake thresholds from the Nordic Nutrition Recommendations 2012 and 2023, no significant difference in iron deficiency was found between individuals consuming above or below 500 g of red meat per week (unadjusted, observed proportions). However, using a lower threshold of 350 g, a higher prevalence of iron deficiency was observed among those consuming less red meat (43.4% vs. 30.9%, *p* = 0.005). Furthermore, among individuals consuming less than one portion of meat (all types) per week, the prevalence of iron deficiency was significantly higher (62.1% vs. 34.2%, *p* < 0.001). Despite these findings, the high prevalence of iron deficiency across all groups suggests that interventions beyond dietary changes may be necessary. There was no difference in anaemia prevalence when comparing the different cut-offs. It should also be noted that red meat intake was estimated by converting reported frequencies into grams, which may not provide the most accurate measurement.

Increased iron deficiency risk was also seen with higher consumption of vegetarian patties and legumes. This may be due to the higher phytate content in these foods [37], or it could be attributed to the reduced meat consumption and not the vegetarian patties per se. Participants who ate moderate amounts of fruit or berries had a lower risk of iron deficiency, which might be due to vitamin C from these foods, which increases the absorption of both heme and non-heme iron [38]. Interestingly, other food groups did not show significant differences in iron deficiency risk, nor did habits of eating breakfast or eating school meals. Additionally, there were no differences in the risk of anaemia across various food group, nor breakfast or school meal consumption.

Several studies [17, 39–42] have showed lower climate, and other environmental impact from vegan and vegetarian diets compared to an omnivorous diet [17], however, we do not have enough information to draw that conclusion. There is an ongoing debate about climate impact from the diet and risk of micronutrient deficiencies [43]. A recent study from Sweden has shown that diets with a lower climate impact does not substantially increase the risk of deficiencies [44], however this study included middle aged people in the 1990s and did not include ferritin as a biomarker.

### Role of iron supplementation and dietary diversity

It is known that iron stores are depleted during periods of growth [45], but the effects and consequences of depleted iron stores for a restricted period is not known and should be investigated further. Interestingly, the study also found that iron supplementation did not fully counteract the lower ferritin levels observed in non-meat-eaters. However, since we did not gather information on the type or dose of iron supplementation, we were unable to explore this issue further. The results from the study highlight the complexity of iron bioavailability and suggest that other dietary factors, such as phytates, which inhibit iron absorption, may also play a significant role [37]. The more restricted diet the more common it was to consume iron containing supplements, 20.6% of vegetarian/vegan participants compared to 8.7% of omnivores. This underscores the need for a holistic approach in dietary recommendations that not only promotes iron-rich foods but also considers factors enhancing iron absorption, such as fruit and vegetables containing Vitamin C. Also, we observed that a lower BMI was associated with lower iron deficiency, which underscores the need to eat enough food. Also, heavy menstrual bleeding as assessed by the SAMANTA score was closely associated with iron deficiency. Even after adjustment for those factors the associations between diet and iron deficiency remained.

### Implications for public health and policy

Given the high prevalence of iron deficiency observed, there is a significant implication for public health strategies. It is crucial for health authorities to develop targeted dietary recommendations that accommodate the dual goals of environmental sustainability and nutritional adequacy. Encouraging the consumption of plant-based diets rich in iron, such as whole grain, nuts and legumes, alongside education on enhancing iron absorption (e.g., consuming vitamin C-rich foods with meals), could form a cornerstone of such strategies. The 'meat factor' enhances the absorption of non-heme iron from meals including red meat, poultry and fish, particularly those containing inhibitors like phytate. The exact reason is uncertain, but one possible explanation is that partially digested peptides, along with cysteine and histidine residues from muscle proteins, bind to non-heme iron, creating soluble complexes that are more easily absorbed [34, 46]. The effect of the 'meat factor' is particularly notable in meals that include inhibitors of iron absorption, such as phytate. Incorporating small amounts of meat, poultry, or fish in such meals can contribute to better iron absorption and may help in preventing iron deficiency. It needs to be emphasised that diets with a higher proportion of plant-based foods and less animal-sourced food might give long-term health benefits [18, 47, 48]. Thus, it is important to continue the shift towards more plant-based dietary habits, both for health and environmental reasons. Moreover, the findings might call for regular screening for iron deficiency in teenage populations, particularly as dietary patterns shift. It is worth noting that anaemia screening based solely on haemoglobin levels may not provide a complete picture, as it does not capture early stages of iron deficiency. Including ferritin measurements in screening protocols could enhance the identification of iron deficiency and improve the effectiveness of interventions. Public health campaigns should also emphasize the importance of balanced eating patterns and not just the reduction of meat consumption for environmental reasons alone.

### Strengths and limitations

This study boasts several strengths. It is one of the largest investigations into iron deficiency among teenage girls in the Nordic countries. Additionally, data were gathered from schools across two cities, enhancing the generalizability of the results. The combination of survey data and objective measures provides a comprehensive view of the participants, offering more insights than using a single method. Furthermore, the survey covered not only dietary habits but also background variables and details about menstruation bleedings. A key strength of this study is the ability to examine diet while accounting for menstrual bleeding adjustments.

However, some limitations should be noted. The population cannot be considered a random sample, as participation was self-selected. This introduces potential recruitment bias, as some girls may have enrolled due to concerns about having an iron deficiency and a desire to confirm it. Another limitation is that during data collection in Malmö, height and weight measurements were not conducted privately. This lack of privacy led to a significant proportion of participants opting out of the measurements, resulting in missing BMI data for 78 participants (16.4%). Additionally, during blood sample collection, a small number of girls fainted in view of others in the queue, leading some to leave and decreasing the number of participants. Participants were asked about recent infections and excluded based on self-reporting, which may not be as reliable as measuring levels of inflammatory markers, such as C-reactive protein (CRP). Ferritin, as an acute-phase protein, can be elevated in response to inflammation, potentially leading to an overestimation of iron stores in individuals with underlying inflammatory conditions. Incorporating inflammatory markers like CRP or interleukin-6 (IL-6) could provide a more accurate assessment of iron status by distinguishing between inflammation-induced and true iron deficiency. Regarding the dietary data: the FFQ used in this study did not aim to cover the participants’ entire diet, making it impossible to estimate the nutritional iron content or other relevant nutrients, as well as energy intake. Although the full FFQ used has not been validated in full, questions in the FFQ been validated to reflect diet quality on a group level [49]. Additionally, reliance on self-reported dietary intake could introduce bias due to potential underreporting or misreporting of food consumption. In Riksmaten Adolecents, it was observed that girls born outside of Sweden had higher risk of iron deficiency. It is a limitation that we did not have access to this information about the participants. Moreover, the cross-sectional design limits the ability to draw causal inferences between diet and iron status. Longitudinal studies would be beneficial to observe the effects of changing dietary patterns over time on micronutrient status.

### Future research

In this study only a small proportion of the participants reported being vegans, which forced us to merge vegans with vegetarians in the analyses. It would be interesting to assess iron deficiency in a population with a higher number of vegans, to be able to analyse the groups separately. Future studies should examine the risk of insufficiency beyond micronutrient intake, focusing on the assessment of bioavailability and nutrient status in various dietary patterns, such as vegan and lacto-ovo-vegetarian diets. The role of BMI in relation to ferritin levels also needs to be investigated further.

## Conclusion

In conclusion, this study showed that diet containing less animal sourced foods was associated with a higher risk of iron deficiency but not anaemia. It highlights the crucial need for tailored nutritional guidance to prevent iron deficiency among Swedish teenage girls, considering both their health and the environmental impacts of their dietary choices. As global dietary shifts continue to evolve, ongoing research and adaptive public health strategies will be essential to ensure the nutritional well-being of future generations.

## Supplementary Information

Below is the link to the electronic supplementary material.

Supplementary file1 (DOCX 816 kb)

## Data Availability

Data described in the manuscript can be made available upon request pending application and approval by the chair of the steering committee for the cohort.

## Abbreviations

BMI: Body mass index
FFQ: Food frequency questionnaire
Hb: Haemoglobin
WHO: World Health Organization
