# Correction: Iron insight: exploring dietary patterns and iron deficiency among teenage girls in Sweden

**DOI:** 10.1007/s00394-025-03679-w

**Published:** 2025-07-05

**Authors:** Anna Stubbendorff, Beata Borgström Bolmsjö, Tomas Bejersten, Eva Warensjö Lemming, Susanna Calling, Moa Wolff

**Affiliations:** 1 Nutritional Epidemiology, Department of Clinical Sciences Malmö, Lund University, Jan Waldenströms gata 35, Malmö, 214 28 Sweden; 2 Center for Primary Health Care Research, Department of Clinical Sciences, Lund University, Malmö, Sweden; 3 University Clinic Primary Care Skåne, Region Skåne, Sweden; 4 Department of Food Studies, Nutrition and Dietetics, Uppsala University, Uppsala, Sweden; 5 Medical Epidemiology, Department of Surgical Sciences, Uppsala University, Uppsala, Sweden

**Correction: European Journal of Nutrition (2025) 64:107**

10.1007/s00394-025-03630-z

In the original version of this article, the columns header “Yes” and “No” in table 1, has moved around both for “Iron deficiency” and “Anemia”. Under “Iron deficiency” the columns header should have read “NO” and “Yes” and under “Anemia” the columns header should have read “NO” and “Yes”

In table 1, last entry of the first column was incorrectly read as “> 500 g fruit and vegetables/day” but should have read “< 500 g fruit and vegetables/day”.

In table 3, last entry of the first column was incorrectly read as “> 500 g fruit and vegetables/day” but should have read “< 500 g fruit and vegetables/day”.

In table 4, the estimated mean differences for haemoglobin were incorrect. The correct table 4 should have appeared as shown below.

**Table 4:**
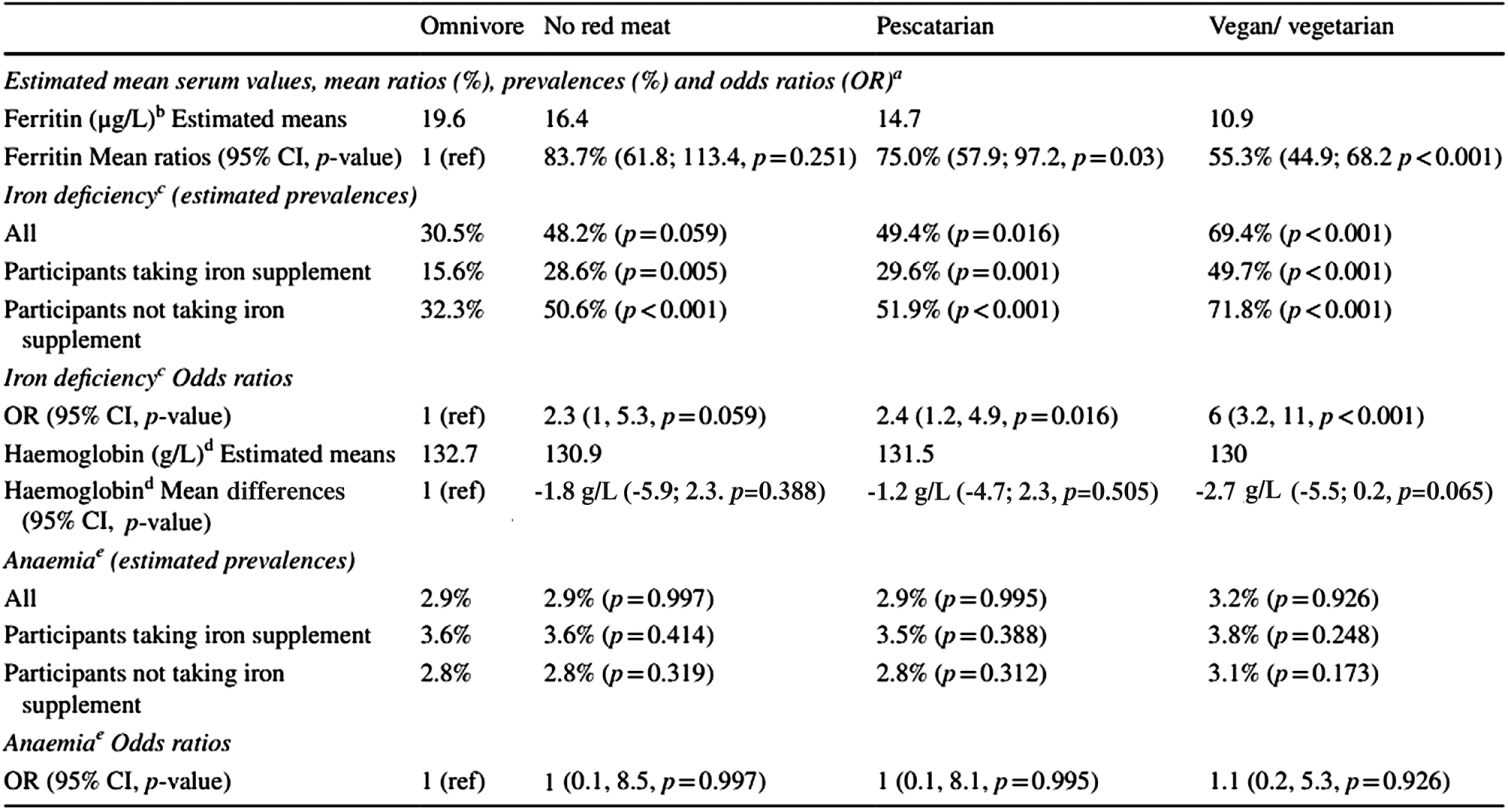
Comparison of self-reported diets and serum levels of ferritin and haemoglobin

Table 4 and 6 of the Supplementary file, estimated mean differences for haemoglobin were incorrect. The correct supplementary file has now been uploaded.

## Electronic supplementary material

Below is the link to the electronic supplementary material.

Supplementary Material 1

