# Supplementary material for: Correction: Iron insight: exploring dietary patterns and iron deficiency among teenage girls in Sweden
Source: Eur J Nutr. 2025 Jul 5;64(5):235. doi: 10.1007/s00394-025-03679-w (PMC12228584; doi:10.1007/s00394-025-03679-w)
Supplement: Supplementary file 1 — Supplementary Material 1 [file 394_2025_3679_MOESM1_ESM.docx]

**Online supplemental material**

Stubbendorff et al. “Iron Insight: Exploring Dietary Patterns
and Iron Deficiency Among Teenage Girls in Sweden”

**Supplemental figure 1.** Part of questionnaire used for data collection about food consumption in the Iron insight study.
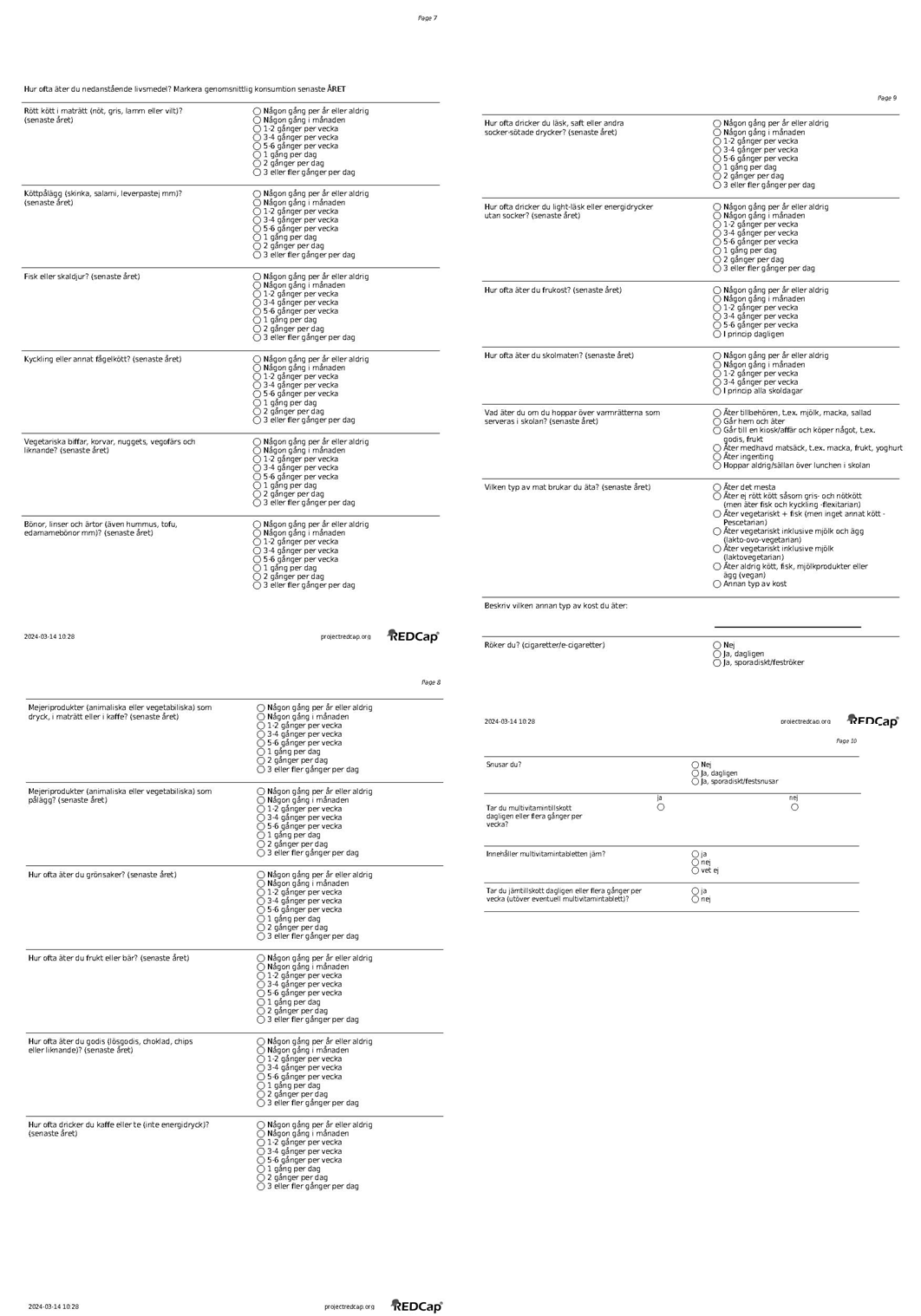


**Supplemental figure 2.** Directed acyclic graph (DAG) of our interpretation of the relationship between the exposure, the outcome, and the covariates.


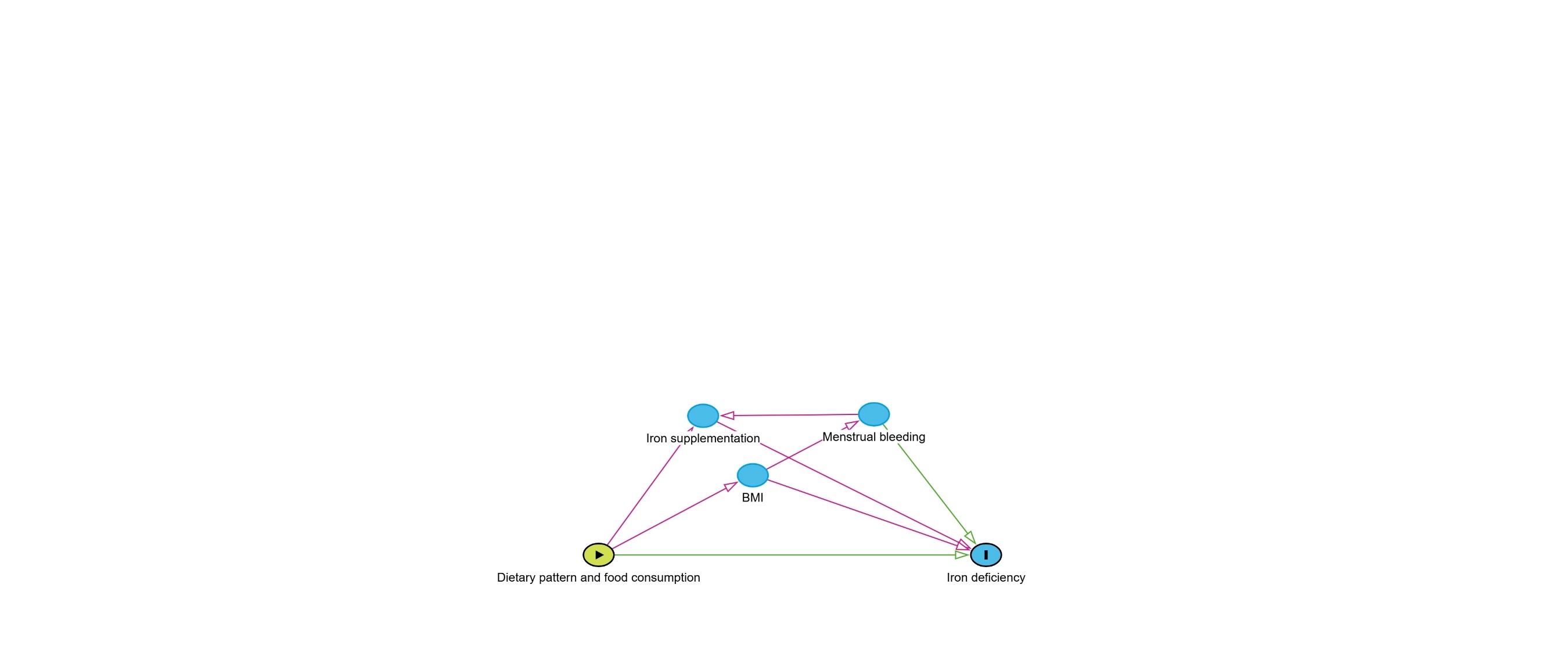


**Supplemental figure 3.** Flowchart of included individuals.


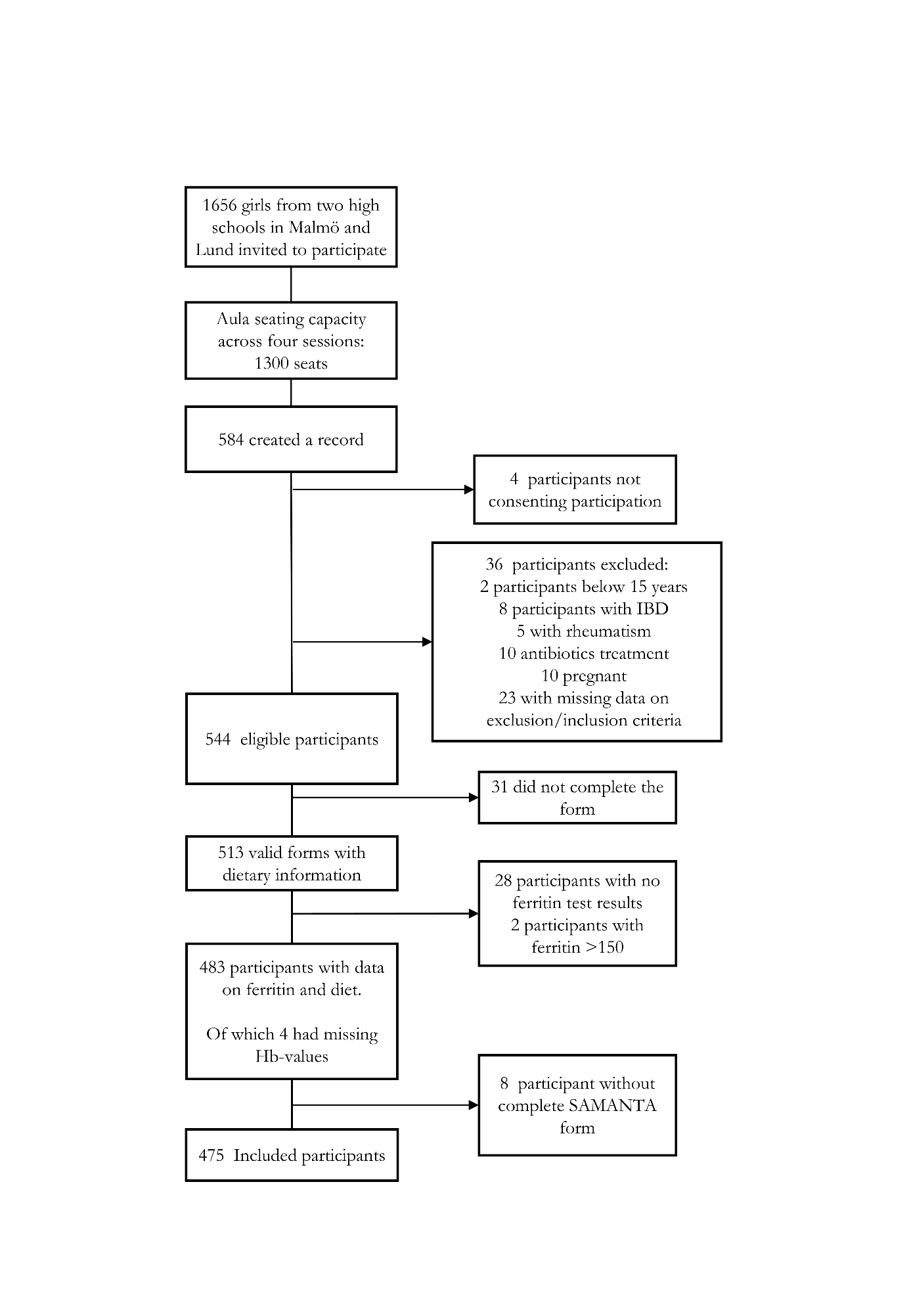


**Supplemental table 1.** Conversion of food consumption frequencies into g/day.

|  |  | **Frequency** | | | | | | | |
| --- | --- | --- | --- | --- | --- | --- | --- | --- | --- |
| **Food consumption during the past year** | grams per serving | Once a year or never | Sometime a month | 1-2 times per week | 3-4 times per week | 5-6 times per week | 1 time per day | 2 times per day | 3 or more times per day |
|  |  |  |  |  |  |  |  |  |  |
| *grams per serving times frequency* |  | *0* | *2/30* | *6/30* | *3.5/7* | *5.5/7* | *1* | *2* | *3* |
|  |  |  |  |  |  |  |  |  |  |
| Red meat in a dish (beef, pork, lamb or game)? | 100 | 0 | 6.7 | 20 | 50 | 78.6 | 100 | 200 | 300 |
| Meat toppings (ham, salami, liver pate, etc.)? | 15 | 0 | 1.0 | 3 | 7.5 | 11.8 | 15 | 30 | 45 |
| Fish or seafood? | 125 | 0 | 8.3 | 25 | 62.5 | 98.2 | 125 | 250 | 375 |
| Chicken or other poultry? | 125 | 0 | 8.3 | 25 | 62.5 | 98.2 | 125 | 250 | 375 |
| Vegetarian steaks, sausages, nuggets, veggie mince etc.? | 100 | 0 | 6.7 | 20 | 50 | 78.6 | 100 | 200 | 300 |
| Beans, lentils and peas (also hummus, tofu, edamame beans, etc.)? | 70 | 0 | 4.7 | 14 | 35 | 55.0 | 70 | 140 | 210 |
| Dairy products (cows milk or plant-based) as a drink, in a dish or in coffee? | 100 | 0 | 6.7 | 20 | 50 | 78.6 | 100 | 200 | 300 |
| Dairy products (cows milk or plant-based) as toppings? | 15 | 0 | 1.0 | 3 | 7.5 | 11.8 | 15 | 30 | 45 |
| How often do you eat vegetables? | 70 | 0 | 4.7 | 14 | 35 | 55.0 | 70 | 140 | 210 |
| How often do you eat fruit or berries? | 100 | 0 | 6.7 | 20 | 50 | 78.6 | 100 | 200 | 300 |
| How often do you eat sweets and snacks (candy, chocolate, chips etc.)? | 90 | 0 | 6.0 | 18 | 45 | 70.7 | 90 | 180 | 270 |
| How often do you drink coffee or tea (not energy drinks)? | 200 | 0 | 13.3 | 40 | 100 | 157.1 | 200 | 400 | 600 |
| How often do you drink soda, juice or other sugar-sweetened beverages? | 250 | 0 | 16.7 | 50 | 125 | 196.4 | 250 | 500 | 750 |
| How often do you drink diet soda or energy drinks without sugar? | 250 | 0 | 16.7 | 50 | 125 | 196.4 | 250 | 500 | 750 |
|  |  |  |  |  |  |  |  |  |  |
| Portion sizes were based on Swedish reference tables (“Vikttabell” and “Mått för mat” [26, 27]), and adopted for this specific project. | | | | | | | | | |

**Supplemental table 2.** Inconsistencies between reported self-identified diet and reported food consumption in the Iron Insight study marked in red.

|  | Vegan | Lacto-vegetarian | Lacto-ovo-vegetarian | Pescatarian | No red meat | Omnivore |
| --- | --- | --- | --- | --- | --- | --- |
| **Self-reported food intake^1^** |  |  |  |  |  |  |
| Red meat (g/day) | 0.0 | 0.8 | 2.8 | 3.8 | 15.3 | 49.2 |
| Processed meat (g/day) | 0.0 | 0.0 | 0.1 | 0.2 | 3.0 | 5.1 |
| Fish (g/day) | 0.0 | 5.2 | 2.5 | 26.3 | 17.0 | 22.5 |
| Poultry (g/day) | 0.0 | 1.0 | 1.0 | 8.2 | 54.3 | 41.5 |
| Dairy in food (g/day) | 16.7 | 119.2 | 119.7 | 114.7 | 92.3 | 98.6 |
| Dairy on bread (g/day) | 5.0 | 12.7 | 11.3 | 9.8 | 9.6 | 8.3 |
|  |  |  |  |  |  |  |
| 1. Conversion of food consumption frequencies into g/day is reported in supplemental table 1. | | | | | | |

**Supplemental table 3.** Participants’ demographics and food intake according to iron deficiency and anaemia.

| **Baseline characteristics in the two schools^1^** | | | |
| --- | --- | --- | --- |
|  | Lund | Malmö | P-value |
| n | 195 (41.1%) | 280 (58.9%) |  |
| Ferritin 15 μg/L | 22.1 (17.9) | 23.2 (16.0) | 0.447 |
| Hemoglobin g/L | 131.1 (10.9) | 132.9 (10.2) | 0.069 |
| Age | 16.6 (1.0) | 16.5 (0.9) | 0.226 |
| High school branch |  |  |  |
| Arts (music, theatre, form) | 33 (17.6%) | 96 (34.8%) | <0.001 |
| Humanities or Social Sciences | 57 (30.3%) | 120 (43.5%) |  |
| Natural sciences | 98 (52.1%) | 60 (21.7%) |  |
| Place of residence |  |  |  |
| In a city | 123 (63.1%) | 219 (78.5%) | <0.001 |
| In a smaller town | 56 (28.7%) | 51 (18.3%) |  |
| In a rural area on the countryside | 16 (8.2%) | 9 (3.2%) |  |
| BMI (mean) | 21.5 (2.6) | 21.8 (2.7) | 0.215 |
| <18,5 | 21 (10.8%) | 21 (7.5%) | <0.001 |
| 18.5-24 | 146 (74.9%) | 164 (58.6%) |  |
| >25 | 19 (9.7%) | 27 (9.6%) |  |
| Unknown | 9 (4.6%) | 68 (24.3%) |  |
| Smoking cigarettes |  |  |  |
| No | 154 (79.0%) | 183 (65.4%) | 0.005 |
| Yes, sporadically | 39 (20.0%) | 91 (32.5%) |  |
| Yes, daily | 2 (1.0%) | 6 (2.1%) |  |
| Using snuff |  |  |  |
| No | 166 (85.1%) | 227 (81.1%) | 0.488 |
| Yes, sporadically | 17 (8.7%) | 29 (10.4%) |  |
| Yes, daily | 12 (6.2%) | 24 (8.6%) |  |
| SAMANTA score total (0-10) | 3.7 (2.9) | 3.3 (2.8) | 0.122 |
| 3 points or more | 114 (58.5%) | 141 (50.4%) | 0.081 |
| Multivitamin users | 33 (17.0%) | 48 (17.2%) | 0.956 |
| Iron supplementation^3^ | 25 (12.8%) | 27 (9.6%) | 0.275 |
| Self-reported diet last year |  |  |  |
| Vegan | 2 (1.0%) | 1 (0.4%) | 0.048 |
| Lacto-vegetarian | 1 (0.5%) | 7 (2.5%) |  |
| Lacto-ovo-vegetarian | 29 (14.9%) | 23 (8.2%) |  |
| Pescatarian | 11 (5.6%) | 27 (9.6%) |  |
| No red meat | 13 (6.7%) | 14 (5.0%) |  |
| Omnivore | 139 (71.3%) | 208 (74.3%) |  |
| Eating breakfast every day | 100 (51.5%) | 153 (54.8%) | 0.480 |
| Eating school meals every school day | 159 (81.5%) | 221 (79.2%) | 0.532 |
| Red meat, g/day^4^ | 34.8 (39.7) | 39.7 (48.0) | 0.239 |
| Processed meat, g/day^4^ | 4.0 (5.5) | 3.9 (5.0) | 0.932 |
| Fish, g/day^4^ | 17.5 (17.8) | 21.6 (23.6) | 0.041 |
| Poultry, g/day^4^ | 31.3 (32.4) | 36.2 (39.6) | 0.154 |
| Total meat, g/day^4^ | 87.5 (67.7) | 101.4 (89.7) | 0.068 |
| Vegetarian patties, g/day^4^ | 33.6 (46.5) | 31.1 (33.7) | 0.500 |
| Legumes, g/day^4^ | 24.7 (29.6) | 21.8 (23.5) | 0.235 |
| Dairy as drink or in food g/day^4^ | 106.6 (93.9) | 98.3 (86.1) | 0.317 |
| Dairy on bread g/day^4^ | 9.0 (9.2) | 8.9 (8.6) | 0.870 |
| Vegetables, g/day^4^ | 113.4 (56.7) | 96.6 (56.5) | 0.002 |
| Fruit and berries, g/day^4^ | 80.4 (70.8) | 66.1 (57.5) | 0.016 |
| Candy and snacks, g/day^4^ | 34.0 (26.4) | 36.6 (31.2) | 0.343 |
| Coffee and tea, g/day^4^ | 113.7 (147.0) | 146.5 (159.5) | 0.023 |
| Sugar sweetened beverages, g/day^4^ | 49.5 (60.2) | 56.5 (71.0) | 0.267 |
| Drinks w artificial sweeteners g/day^4^ | 52.5 (99.9) | 55.7 (95.7) | 0.724 |
| Red meat <500 g/week | 170 (87.2%) | 225 (80.4%) | 0.051 |
| Red meat <350g/week | 111 (56.9%) | 163 (58.2%) | 0.779 |
| All meat <1 port/week (100 g)^5^ | 34 (17.4%) | 32 (11.4%) | 0.063 |
| >500 g fruit and vegetables/day | 188 (96.4%) | 278 (99.3%) | 0.024 |
| ^1.^ Values are mean (SD) for continuous variables, and n (%) for categorical variables | | | |

**Supplemental table 4.** Anaemia analysed with logistic regression for consumption of food groups, adjusted for covariates BMI, SAMANTA score and dietary iron supplementation. Based on 473 individuals.

|  | **Odds ratios for anaemia and food consumption** | | | | |
| --- | --- | --- | --- | --- | --- |
|  | **Portions per** | | | |  |
|  | **month** | **week** | | | **p** |
| **Food groups** | ≤1 | 1-2 | 3-4 | ≥5 |  |
|  |  |  |  |  |  |
| Red meat, n | *143* | *135* | *120* | *76* |  |
|  | 1 (ref) | 0.81 (0.21, 3.14) |  |  | 0.763 |
|  | 1 (ref) |  | 0.49 (0.09, 2.66) |  | 0.411 |
|  | 1 (ref) |  |  | 1.27 (0.28, 5.68) | 0.753 |
| Processed meat, n | *247* | *85* | *77* | *65* |  |
|  | 1 (ref) | 0.57 (0.12, 2.7) |  |  | 0.479 |
|  | 1 (ref) |  | 1 |  | - |
|  | 1 (ref) |  |  | 0.69 (0.15, 3.3) | 0.644 |
| Poultry, n | *138* | *195* | *108* | *34* |  |
|  | 1 (ref) | 0.36 (0.09, 1.48) |  |  | 0.157 |
|  | 1 (ref) |  | 0.67 (0.16, 2.84) |  | 0.588 |
|  | 1 (ref) |  |  | 1.5 (0.28, 8.13) | 0.636 |
| Fish and shellfish, n | *225* | *204* | *39* | *6* |  |
|  | 1 (ref) | 1.65 (0.55, 4.92) |  |  | 0.373 |
|  | 1 (ref) |  | 1 |  | - |
|  | 1 (ref) |  |  | 1 | - |
| Vegetarian patties, n | 180 | 141 | 84 | 69 |  |
|  | 1 (ref) | 0.31 (0.06, 1.47) |  |  | 0.139 |
|  | 1 (ref) |  | 0.55 (0.11, 2.67) |  | 0.457 |
|  | 1 (ref) |  |  | 0.6 (0.12, 2.94) | 0.527 |
| Legumes, n | 152 | 162 | 94 | 67 |  |
|  | 1 (ref) | 0.3 (0.06, 1.49) |  |  | 0.140 |
|  | 1 (ref) |  | 0.49 (0.09, 2.49) |  | 0.386 |
|  | 1 (ref) |  |  | 1.42 (0.38, 5.27) | 0.604 |
| Dairy products in food or as drink, n | *52* | *71* | *81* | *271* |  |
|  | 1 (ref) | 0.2 (0.02, 2.01) |  |  | 0.171 |
|  | 1 (ref) |  | 0.2 (0.02, 1.98) |  | 0.168 |
|  | 1 (ref) |  |  | 0.59 (0.15, 2.31) | 0.450 |
| Dairy on bread, n | *95* | *110* | *87* | *182* |  |
|  | 1 (ref) | 0.31 (0.03, 3.05) |  |  | 0.314 |
|  | 1 (ref) |  | 0.8 (0.13, 5.01) |  | 0.807 |
|  | 1 (ref) |  |  | 1.69 (0.43, 6.72) | 0.454 |
| Vegetables | 5 | 22 | 47 | 400 |  |
|  | 1 (ref) | 1.24 (0.15, 10.39) |  |  | 0.84 |
|  | 1 (ref) |  | 0.66 (0.08, 5.21) |  | 0.69 |
|  | 1 (ref) |  |  | 1 | - |
| Fruit and berries, n | *54* | *101* | *106* | *214* |  |
|  | 1 (ref) | 0.42 (0.09, 1.98) |  |  | 0.274 |
|  | 1 (ref) |  | 0.27 (0.05, 1.54) |  | 0.139 |
|  | 1 (ref) |  |  | 0.33 (0.08, 1.31) | 0.115 |
| Candy and snacks, n | *35* | *217* | *164* | *58* |  |
|  | 1 (ref) | 0.98 (0.11, 8.76) |  |  | 0.987 |
|  | 1 (ref) |  | 0.68 (0.07, 6.93) |  | 0.743 |
|  | 1 (ref) |  |  | 2.37 (0.24, 22.93) | 0.458 |
| Coffee and tea, n | *140* | *89* | *70* | *175* |  |
|  | 1 (ref) | 0.46 (0.09, 2.39) |  |  | 0.358 |
|  | 1 (ref) |  | 0.31 (0.04, 2.66) |  | 0.285 |
|  | 1 (ref) |  |  | 0.71 (0.21, 2.42) | 0.589 |
| Sugar sweetened beverages (SSB), n | *208* | *182* | *61* | *22* |  |
|  | 1 (ref) | 0.92 (0.27, 3.17) |  |  | 0.900 |
|  | 1 (ref) |  | 1.63 (0.38, 6.94) |  | 0.507 |
|  | 1 (ref) |  |  | 1 | - |
| Light drinks, n | *278* | *104* | *56* | *36* |  |
|  | 1 (ref) | 1.38 (0.4, 4.77) |  |  | 0.615 |
|  | 1 (ref) |  | 0.56 (0.07, 4.68) |  | 0.593 |
|  | 1 (ref) |  |  | 0.98 (0.12, 8.3) | 0.988 |
|  |  |  |  |  |  |
|  | | | | | |

**Supplemental table 5.** Sensitivity analyses comparison of self-reported diets and serum levels of ferritin and haemoglobin, without adjusting for covariates.

| **Estimated mean serum values, mean ratios (%), prevalences (%) and odds ratios (OR)^1^** | | | | |
| --- | --- | --- | --- | --- |
|  | **Omnivore** | **No red meat** | **Pescatarian** | **Vegan/ vegetarian** |
|  |  |  |  |  |
| Ferritin ( μg/L)^2^  Estimated means | 18.9 | 14.8 | 14.9 | 11 |
| Ferritin  Mean ratios (95% CI, p-value) | 1 (ref) | 78.4% (57.1, 107.7, p=0.133) | 78.7% (60, 103.2, p=0.083) | 58.3% (46.9, 72.5, p<0.001) |
|  |  |  |  |  |
| **Iron deficiency^3^**  **(estimated prevalences)** |  |  |  |  |
| All | 30.8% | 51.9% (p<0.001) | 47.4% (p<0.001) | 66.7% (p<0.001) |
|  |  |  |  |  |
| **Iron deficiency^3^**  **Odds ratios** |  |  |  |  |
| OR (p-value) | 1 (ref) | 2.4 (1.1, 5.3, p=0.028) | 2 (1, 4, p=0.042) | 4.5 (2.5, 7.9, p<0.001) |
|  |  |  |  |  |
|  |  |  |  |  |
|  |  |  |  |  |
| Haemoglobin (g/L)^4^  Estimated means | 132.6 | 130.5 | 131.8 | 130.3 |
| Haemoglobin^4^  Mean differences (95% CI, p-value) | 1 (ref) | -2.1 g/L (-6.3, 2, p=0.308) | -0.8 g/L (-4.4, 2.7, p=0.641) | -2.3 g/L (-5.2, 0.5, p=0.113) |
|  |  |  |  |  |
| **Anaemia^5^**  **(estimated prevalences)** |  |  |  |  |
| All | 2.9% | 3.7% (p=0.308) | 2.6% (p=0.311) | 3.2% (p=0.151) |
|  |  |  |  |  |
| **Anaemia^5^**  **Odds ratios** |  |  |  |  |
| OR (95% CI, p-value) | 1 (ref) | 1.3 (0.2, 10.5, p=0.81) | 0.9 (0.1, 7.3, p=0.928) | 1.1 (0.2, 5.2, p=0.886) |
|  |  |  |  |  |
| 1. Models are based on logistic regression for categorical variables and ANOVA for continuous variables. P-values are the pairwise test using omnivores as reference.  2. Ferritin was modelled on the log-scale and geometric means are presented.  3. Ferritin below 15 μg/L  4. Two participants have missing values on anaemia (n=473).  5. Haemoglobin below 110 g/L if participant age <19 years old and 117 g/L for participants ≥19 years. | | | | |

**Supplemental table 6.** Comparison of self-reported diets and serum levels of ferritin and haemoglobin, excluding participants with missing data on BMI (n=398).

| **Estimated mean serum values, mean ratios (%), prevalences (%) and odds ratios (OR)^1^** | | | | |
| --- | --- | --- | --- | --- |
|  | **Omnivore** | **No red meat** | **Pescatarian** | **Vegan/ vegetarian** |
|  |  |  |  |  |
| Ferritin ( μg/L)^2^  Estimated means | 20.1 (18.3, 21.9) | 15.3 (10.4, 20.1) | 15.4 (11.1, 19.6) | 11 (8.7, 13.2) |
| Ferritin  Mean ratios (95% CI, p-value) | 1 (ref) | 76.1% (54.8, 105.6, p=0.102) | 76.6% (57.4, 102.3, p=0.071) | 54.7% (43.8, 68.4, p=0) |
|  |  |  |  |  |
| **Iron deficiency^3^**  **(estimated prevalences)** |  |  |  |  |
| All | 28.7% | 53.4% (p=0.017) | 49.1% (p=0.019) | 70.4% (p<0.001) |
| Participants taking iron supplement | 13.5% | 31.4% (p=0.008) | 27.7% (p=0.007) | 48.9% (p<0.001) |
| Participants not taking iron supplement | 30.7% | 56.2% (p<0.001) | 51.8% (p<0.001) | 73.1% (p<0.001) |
|  |  |  |  |  |
| **Iron deficiency^3^**  **Odds ratios** |  |  |  |  |
| OR (95% CI, p-value) | 1 (ref) | 3.1 (1.2, 7.9, p=0.017) | 2.6 (1.2, 5.7, p=0.019) | 6.9 (3.6, 13.4, p=0) |
|  |  |  |  |  |
|  |  |  |  |  |
|  |  |  |  |  |
| Haemoglobin (g/L)^4^  Estimated means | 132.9 (131.7, 134) | 130.4 (126.3, 134.5) | 131.9 (128.3, 135.5) | 129.3 (126.6, 132) |
| Haemoglobin^4^  Mean differences (95% CI, p-value) | 1 (ref) | -2.4 g/L (-6.7, 1.8, p=0.261) | -1 g/L (-4.8, 2.8, p=0.597) | -3.6 g/L (-6.5, -0.6, p=0.017) |
|  |  |  |  |  |
| **Anaemia^5^**  **(estimated prevalences)** |  |  |  |  |
| All | 2.4% | 3.3% (p=0.779) | 4% (p<0.001) | 0% (p=0.54) |
| Participants taking iron supplement | 2.5% | 3% (p=0.354) | 0% (p<0.001) | 0% (p<0.001) |
| Participants not taking iron supplement | 2.5% | 3.4% (p=0.32) | 4.1% (p=0.166) | 1.8% (p=0.343) |
|  |  |  |  |  |
| **Anaemia^5^**  **Odds ratios** |  |  |  |  |
| OR (95% CI, p-value) | 1 (ref) | 1.4 (0.2, 12.5, p=0.779) | 1 (0, 0, p=0) | 1.7 (0.3, 8.6, p=0.54) |
|  |  |  |  |  |
| 1. Models are based on logistic regression for categorical variables and ANOVA for continuous variables. All analyses are adjusted for BMI, SAMANTA score and iron supplementation. P-values are the pairwise test using omnivores as reference.  2. Ferritin was modelled on the log-scale and geometric means are presented.  3. Ferritin below 15 μg/L  4. Two participants have missing values on anaemia (n=396).  5. Haemoglobin below 110 g/L if participant age <19 years old and 117 g/L for participants ≥19 years. | | | | |
